# Bixafen, Prothioconazole, and Trifloxystrobin Alone or in Combination Have a Greater Effect on Health Related Gene Expression in Honey Bees from Nutritionally Deprived than from Protein Supplemented Colonies

**DOI:** 10.3390/insects15070523

**Published:** 2024-07-11

**Authors:** Aline Y. Kato, Tainá A. L. Freitas, Cássia R. A. Gomes, Thais R. R. Alves, Yara M. M. Ferraz, Matheus F. Trivellato, David De Jong, Jaqueline D. Biller, Daniel Nicodemo

**Affiliations:** 1 Post Graduate Program in Animal Science, School of Agricultural and Veterinarian Sciences, São Paulo State University (Unesp), Jaboticabal 14884-900, SP, Brazil; 2 Genetics Department, Ribeirão Preto Medical School, University of São Paulo, Ribeirão Preto 14049-900, SP, Brazil; 3 Department of Animal Science, College of Agricultural and Technology Sciences, São Paulo State University (Unesp), Dracena 17915-899, SP, Brazil; 4 Department of Animal Science, School of Agricultural and Veterinarian Sciences, São Paulo State University (Unesp), Jaboticabal 14884-900, SP, Brazil

**Keywords:** contact exposure, detoxification, fungicide, immunity, nutrition, RT-PCR

## Abstract

**Simple Summary:**

In many regions of the world, foraging bees encounter an environment with an inadequate supply of nutrients compared with the colonies’ demands, as well as exposure to pesticides used to combat plant pests and diseases, including fungicides which historically were considered innocuous for bees. The use of pesticide mixtures can increase the risk for bees, especially when limited foraging leads to nutritional deficiencies. In our study, we compared the effects of the commercial fungicides bixafen, prothioconazole, and trifloxistrobin, alone and in combination, on foraging honey bees that were collected from colonies with supplemented or restricted food availability. For this purpose, we assessed the expression of health-related genes, including antioxidant genes (SOD-1, CAT, and GPX-1), detoxification genes (GST-1 and CYP306A1), the storage protein gene vitellogenin, and immune system antimicrobial peptide genes (defensin-1, abaecin, hymenoptaecin, and apidaecin) using real-time PCR. All three fungicides caused alterations in the expression of these genes, especially the mixture with the three active ingredients, both in bees from colonies with supplemented feeding and those from colonies subjected to food restriction. The effect of fungicide exposure on gene expression was dependent on bees’ nutritional status, which suggests that adequate nutrition can help counteract the effects of fungicides on bees.

**Abstract:**

The aim of this study was to evaluate whether alterations in food availability compromise the metabolic homeostasis of honey bees exposed to three fungicides alone or together. Ten honey bee colonies were used, with half receiving carbohydrate-protein supplementation for 15 weeks while another five colonies had their protein supply reduced with pollen traps. Subsequently, forager bees were collected and exposed by contact to 1 or 7 µg of bixafen, prothioconazole, or trifloxystrobin, either individually or in combination. After 48 h, bee abdomens without the intestine were used for the analysis of expression of antioxidant genes (SOD-1, CAT, and GPX-1), detoxification genes (GST-1 and CYP306A1), the storage protein gene vitellogenin, and immune system antimicrobial peptide genes (defensin-1, abaecin, hymenoptaecin, and apidaecin), through real-time PCR. All fungicide treatments induced changes in gene expression, with bixafen showing the most prominent upregulation. Exposure to 1 µg of each of the three pesticides resulted in upregulation of genes associated with detoxification and nutrition processes, and downregulation of immune system genes. When the three pesticides were combined at a dose of 7 µg each, there was a pronounced downregulation of all genes. Food availability in the colonies affected the impact of fungicides on the expression of the studied genes in forager bees.

## 1. Introduction

Bees are susceptible to numerous environmental stressors, including pesticides; among these there is little or no use restriction for fungicides in many countries [1,2]. This is of concern because fungicides account for approximately 35% of the global pesticide market [3]. The widespread use of fungicides to control and prevent damage due to pathogenic fungi often results in the development of fungicide-resistant strains, implying the need for new plant disease control strategies. An alternative to help overcome resistance has been to apply fungicide mixtures to combat one or more pathogens at various stages of the life cycle [4].

Lethal effects for bees resulting from exposure to fungicides are typically observed through exposure to relatively high doses (>100 μg/bee) [5,6,7]. However, the actual impact on bee health needs to be evaluated with environmentally relevant doses of fungicides. Data on these doses can be difficult to obtain, especially for new products, for which there is little information concerning contamination levels on treated crops. Determining impact is more complicated when multiple active ingredients (a.i.) with fungicidal action are used in the same commercial product. These mixtures can lead to synergistic effects, which may not be observed when they are evaluated individually [8,9,10].

A commercial product that contains the active ingredients bixafen (bix), prothioconazole (pro), and trifloxystrobin (tri) is recommended for controlling fungal diseases in various major crops such as cotton, corn, sunflower, and soybean, with no restrictions on applications during the flowering period of these plants [11]. Since these crops are visited by bees for pollen and nectar, these non-target organisms may be exposed to the fungicidal active ingredients by contact or ingestion of contaminated food [12,13]. In Brazil, in 2022, 1981 t of bix, 5193 t of pro and 5195 t of tri were commercialized in various commercial fungicides containing one or more of these a.i. [14].

Bix is a fungicide belonging to the chemical group of carboxamides; it interferes with the mitochondrial respiratory chain by inhibiting the enzyme succinate dehydrogenase, which is responsible for electron transfer in complex II. Consequently, with less energy available, vital fungal functions are impaired, impacting reproduction and potentially leading to mortality [15,16]. There are few studies addressing the effects of bix on pollinators. Another fungicide from the same carboxamide chemical group, boscalid, is more widespread, and known negative impacts on bee health due to exposure to this fungicide include reduced longevity [17] and decreased wing beat frequency, rendering bees lethargic [18].

The fungicide tri is a member of the strobilurin chemical group, which inhibit mitochondrial respiration in complex III of the respiratory chain. Consequently, the energy cycle within the fungus is interrupted, halting ATP production [19]. Exposure of the Brazilian stingless bee with the common name Mandaçaia (*Melipona quadrifasciata*) to tri combined with the fungicide tebuconazole resulted in increased mortality; additionally, bees were repelled from sprayed tomato flowers. With fewer bees and reduced flower visits, there was a negative impact on fruit production [20].

The fungicide pro belongs to the triazolintione group and interferes with sterol biosynthesis. When pro binds to the enzyme sterol C14-demethylase, it blocks the sterol biosynthesis pathway, resulting in inhibition of production of phospholipids, accumulation of free fatty acids, and fungal death [16]. This a.i. is marketed in over 60 countries and is the third best-selling fungicide worldwide [21]. There is disagreement regarding the level of toxicity of this fungicide for honey bees [22,23]. However, triazole fungicides, which have the same mode of action as the triazolintione group, can affect bee behavior and susceptibility to diseases [24,25].

Another factor inherent to conventional agriculture that can be detrimental to bees is decreased natural food resources. Large-scale monocultures, coupled with deforestation of native vegetation, result in reduced availability of pollen and nectar [26]. Though some crops provide pollen, the amino acid composition of pollen from a single species may not meet the nutritional demands of the bees [27,28], leading to malnutrition and increased susceptibility to environmental stressors [29,30].

Our objective was to determine how individual and mixed fungicide ingredients affect the expression of genes related to honey bee health and whether nutritional condition of the colonies they came from affects gene function. We treated forager bees from well fed and from nutritionally deprived colonies with the fungicides bix, pro, and tri and measured the expression of antioxidant, detoxification, storage protein, and antimicrobial protein genes.

## 2. Material and Methods

### 2.1. Location of the Experiment and Preparation of the Honey Bee Colonies

The study was conducted in the municipality of Dracena, state of São Paulo, at a latitude of 21°27′37″ South and longitude of 51°33′21″ West, and altitude 392 m. The local climate is characterized as tropical with a dry season. During the experiment, the average temperature was 26.9 °C, the average relative humidity was 63.1%, and there was 390 mm of rainfall.

Ten colonies of Africanized honey bees (*Apis mellifera*), headed by open mated queens, and kept in 10 frame Langstroth hives, were used. In the week prior to the start of the experiment, the colonies were standardized so that they presented at that time six frames of brood and four frames of food. They were divided into two groups of five hives: one group with supplemented feeding (SF) and the other with reduced food (RF).

Over a period of 15 weeks, the SF colonies were able to store all the food they collected in the field and were supplemented with 500 mL of syrup containing water and sucrose in equal proportions by weight, twice a week, as well as 100 g of protein paste composed of two parts multifloral bee pollen and one part of honey, provided once a week. Both pollen and honey were obtained from hives in the same apiary where the experiment was conducted. For the other group (RF), during the same period, there was no food supplementation, and pollen traps were installed at the hive entrances to reduce the availability of protein for the bees.

### 2.2. Bee Pollen Collection from Colonies with Reduced Feeding

In the RF group, bee pollen was collected daily and weighed using a semi-analytical balance, recording the quantities obtained weekly for each colony. To determine the efficiency of the pollen collectors (%), the number of forager bees going through the pollen traps was observed during 30-min intervals between 8 and 10 AM, with three repetitions per colony. The number of bees entering with pollen and the number of pollen pellets falling into the pollen trap drawer were recorded. The efficiency of the traps was calculated as the ratio of the number of pollen pellets collected, divided by two times the number of bees entering the collectors with pollen loads.

### 2.3. Exposure of Forager Bees to Single Fungicides or in Combination

Forager bees were collected from the hive entrances after 15 weeks of feeding management. The bees were placed in 0.25 L clear round plastic containers with perforated lids. In the laboratory, the bees were anesthetized in a freezer (−20 °C) for several minutes.

The immobilized bees were exposed by contact to bix, pro, and tri, by applying 0.5 µL of an acetone solution containing 1 or 7 µg of one or all three active ingredients in combination, using a micropipette, on the dorsal part of the thorax of the bees [31]. The doses were determined based on a study estimating the dose at which a bee would be exposed by contact when applying a fungicide containing the three active ingredients according to agronomic recommendations [32]. The acetone solvent alone was applied for the control. Acetone and the fungicide a.i. were purchased from Sigma-Aldrich, St. Louis, MO, USA.

Following contact exposure, groups of 20 forager bees from colonies that received the same feeding management and exposure dose to the fungicides were placed in new plastic containers and fed ad libitum with syrup containing two parts sucrose and one part water (weight/weight), maintained for a period of 48 h in an incubator with controlled temperature (33° ± 1 °C) and humidity (70 ± 10%).

### 2.4. Gene Expression Assessment

After 48 h in the incubator, the bees were killed in a freezer for dissection, which was carried out with the aid of a stereo binocular microscope, fine tip scissors, and entomological forceps. We discarded heads, thoraxes, and intestines, leaving only the abdomens. Each sample consisted of six abdomens, with four samples per treatment (two colony feeding regimes × four fungicides × two doses and controls).

#### 2.4.1. RNA Extraction and cDNA Synthesis

The gene expression analysis was performed by Real-Time qPCR, using the primers described in Table 1.

For RNA extraction, 1 mL of Trizol^®^ LS reagent (Invitrogen™, Waltham, MA, USA) and 200 µL of chloroform were used for each sample of six dissected abdomens (approximately 100 mg). The tubes were centrifuged at 12,000× *g* for 15 min at 4 °C. The upper aqueous phase was transferred to another microtube, to which isopropanol was added. The samples were vortexed and centrifuged at 12,000× *g* for 10 min at 4 °C. Finally, the supernatants were discarded, RNA pellets were washed three times with 100 µL of ethanol, centrifuged, the supernatant discarded, and pellets were air-dried for 15 min. Subsequently, they were suspended in molecular grade water (Sigma, St. Louis, MO, USA).

After RNA isolation, quantification was made in ng by absorbance at 260 nm using a Nanodrop-1000 (Thermo Scientific, Waltham, MA, USA). An aliquot of RNA was processed in a 1% agarose gel and post-stained with GelRed^®^ (Sigma) to verify RNA integrity. The cDNA was constructed from the extracted RNA by reverse transcription using 1 μg of total RNA and iScriptTM III (Life Technologies, Waltham, MA, USA). The resulting cDNA was stored at −20 °C until use. Subsequently, the sequence was amplified by polymerase chain reaction (PCR) using target primers.

#### 2.4.2. Determination of Relative Gene Expression

The determination of gene expression was carried out using the real-time polymerase chain reaction (qPCR-RT) method, and the results were analyzed by relative quantification using the 2^−ΔΔCt^ method [38,39]. qPCR reactions were performed in 96-well plates using iTaqTM Universal SYBR^®^ Green Supermix (Bio-Rad, Hercules, CA, USA). Reactions were conducted following the manufacturer’s instructions, with 20 µL individual reaction mixtures consisting of 10 µL of SYBRGreen PCR Master Mix (2×), 8 µL of 200 nM primers, and 2 µL of cDNA template (samples) [39].

All reactions were performed using the iCycler iQ™ real-time qPCR detection system (Bio-Rad Laboratories) under the following protocol: 95 °C for 5 min, 40 cycles of 95 °C for 30 s, 56 °C for 30 s, followed by analysis of the dissociation curve (melting curve) to verify amplification of a single product. The denaturation-hybridization-synthesis cycle temperatures used were according to Pfaffl et al. (2004) [38].

The threshold of the exponential phase, denoted as the Cycle Threshold (Ct), was detected during the temperature cycles, precisely quantifying the products of the amplification reaction by fluorescence emission. Expression data was used to calculate the Ct values. PCR efficiency and the relative expression ratio of target genes in experimental groups versus control groups were calculated according to the method of Pfaffl et al. (2004) [38]. The comparative Ct method (2^−ΔΔCt^ method) was used to analyze the expression level of these genes relative to the RPL32 gene [33].

### 2.5. Statistical Analyses

The mean amount of bee pollen collected weekly from each colony was calculated for the RF colonies. The efficiency of the pollen traps was evaluated by the mean of 12 observations across four hives. Data dispersion was determined by calculating the standard error.

For the gene expression analysis, a completely randomized design with a factorial scheme was employed. The experiment included two nutritional levels (supplemented and reduced feeding), four fungicides (three active ingredients and a mixture of all three), and three doses (control, 1 µg/bee, and 7 µg/bee). Since the data did not follow a normal distribution, a generalized linear mixed model was used. This model considered feeding status and exposure status as fixed variables and replicates as random effects for each fungicide. The data were then compared using Tukey’s test at a 5% significance level [40].

## 3. Results

### 3.1. Feeding Management of Colonies

During the 15-week feeding management period, each of the five colonies with food supplementation fully consumed the 100 g of protein supplement and the 1000 mL of sucrose syrup offered weekly. One colony from the RF group succumbed at the 11th week and was not replaced. Therefore, to maintain the same number of colonies among the groups, one colony from the SF group was randomly selected and removed from the experiment.

In the RF group, 33.8 ± 4.9 g of bee pollen was collected weekly per colony. By observing foraging bees with pollen in their corbiculae going through the pollen traps, it was estimated that the efficiency of these collectors was 57.9 ± 5.1%. Thus, of all the pollen collected by the bees from colonies with reduced feeding, a mean of only 42.1% remained available for the bees.

### 3.2. Gene Expression

**Figure 1:**
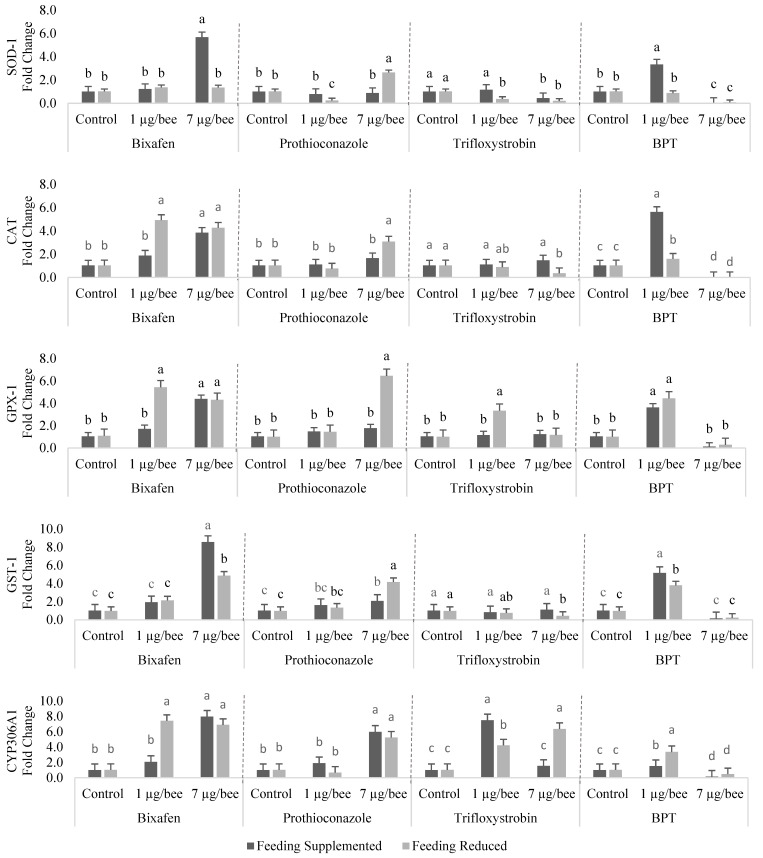
Expression in the fat body of antioxidant and detoxification genes of honey bee foragers from colonies with supplemented or restricted feeding and exposed or not to bixafen (bix), prothioconazole (pro), trifloxystrobin (tri), and a mixture with all three a.i. (BPT). Dose 1 = 1 µg A. I. bee^−1^; dose 7 = 7 µg bee^−1^. Shown are the means of four biological samples per treatment in triplicate. For each gene, means followed by the same letter within the same a.i. or mixture with all three a.i. do not differ significantly from each other (*p* < 0.05), according to the Tukey test.

**Figure 2:**
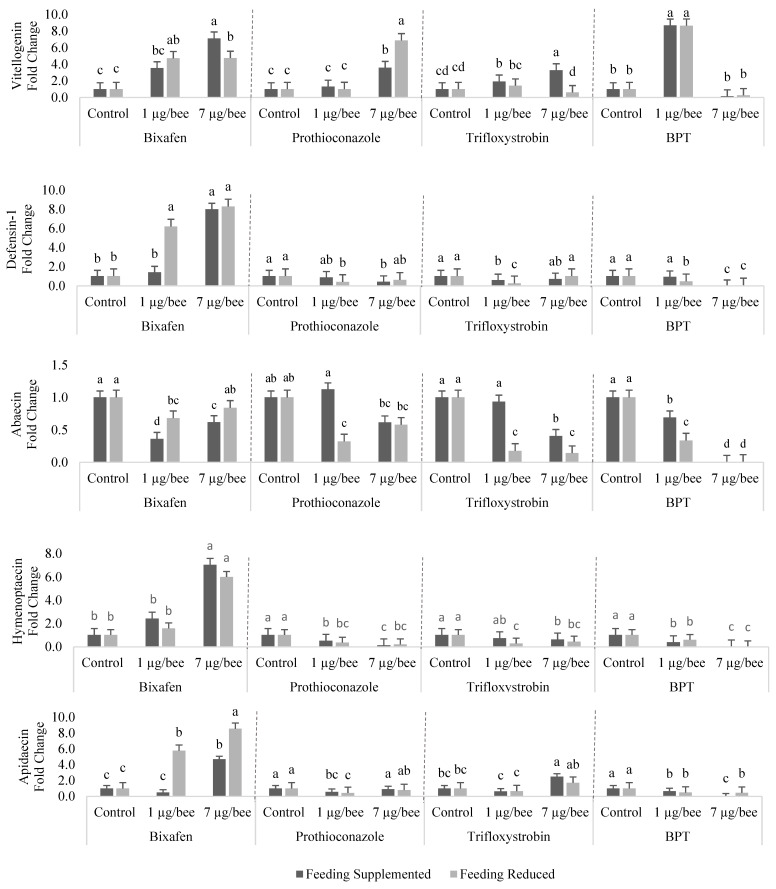
Expression in the fat body of vitellogenin and immune system antimicrobial peptide genes of honey bee foragers from colonies with supplemented or restricted feeding and exposed or not to bixafen (bix), prothioconazole (pro), trifloxystrobin (tri) or a mixture with all three a.i. (BPT). Dose 1 = 1 µg a.i. bee^−1^; dose 7 = 7 µg bee^−1^. Shown are the means of four biological samples per treatment in triplicate For each gene, means followed by the same letter within the same a.i. or mixture with all three a.i. do not differ significantly from each other (*p* < 0.05), according to the Tukey test.

We observed a decrease in activation of all the genes when bees were exposed to 7 µg of each of the three a.i. in combination (BPT), except for GPX-1 and GST-1, for bees from both feeding management groups (Figure 1 and Figure 2). With the lowest dose of each a.i. in combination (1 µg), upregulation of the genes was observed, except for those involved in the immune system, which were downregulated. When comparing the controls of bee samples from SF and RF colonies, no difference in the expression of any of the genes was observed (Figure 1 and Figure 2).

The expression of SOD-1 was increased by exposure to bix (7 µg) in bees from the SF group. With pro, there was a decrease (with 1 µg) and an increase (with 7 µg) in the expression of this gene for bees from the RF group. Tri led to a decrease in the gene expression of SOD-1 with both doses for bees from the RF group and with the higher dose for bees from the SF group. With the mixture of fungicides, the expression of SOD-1 was increased with 1 µg per bee from the SF group and decreased with 7 µg per bee, in bees from both dietary managements (Figure 1).

For the catalase gene, bix promoted upregulation with both doses in both dietary managements, except in bees from the SF group treated with the lower dose. With pro, there was an increase in the expression of this gene with 7 µg in bees from the RF group. For tri at a higher dose, there was a decrease in catalase expression. With the mixture of the three fungicides, an increase and a decrease in the expression of this gene were observed with the lower and higher doses, respectively, in bees from both dietary managements (Figure 1).

The expression of GPX-1 was upregulated with the use of bix, except for exposure to the lower dose, in bees from the SF group. Pro and tri led to an increase in GPX-1 expression with the use of 7 µg and 1 µg per bee from the RF group, respectively. With the mixture of fungicides at the lower dose, there was an increase in the expression of this gene (Figure 1).

There was an increase in the expression of GST-1 when bees from both dietary managements were exposed to the higher dose of both bix and pro. For tri (with 7 µg), there was a decrease in the expression of this gene in bees from the RF group. With BPT, there was upregulation of GST-1 with a dose of 1 µg per bee from both dietary groups (Figure 1).

Upregulation of the CYP306A1 gene was observed with exposure of bees to all fungicides applied individually, except for the lower dose of bix in bees from the SF group, the lower dose of pro in bees from both dietary managements, and the higher dose of tri for bees from the SF group. BPT promoted upregulation and downregulation of CYP306A1 with the use of 1 µg and 7 µg, respectively, of each active ingredient in bees from both dietary managements (Figure 1).

The expression of vitellogenin was upregulated with the exposure of bees to bix, except for the lower dose applied to bees from the SF group. With pro, upregulation was observed with the dose of 7 µg per bee in both dietary managements. For tri (1 µg/bee), vitellogenin was upregulated in bees from the SF group (Figure 2). With BPT at the lower dose, vitellogenin expression increased in bees from both dietary managements.

Defensin-1 was upregulated by exposure to bix, except for bees from the SF group exposed to the lower dose of this active ingredient. With the lower and higher doses of pro, there was downregulation of this gene in bees from the RF and SF groups. For tri and BPT, there was a decrease in the expression of defensin-1, except for the higher dose of tri in bees from both dietary managements and for bees from the SF group exposed to the lower dose of BPT (Figure 2).

The abaecin gene was downregulated when bees from both dietary managements were exposed to bix, except with the application of the higher dose in bees from the RF group. With pro, the only change was observed with the use of 1 µg per bee in the RF group. Tri promoted downregulation of abaecin, except for bees from the SF group exposed to the lower dose. Both doses of BPT resulted in a decrease in the expression of this gene in bees from both dietary managements (Figure 2).

Hymenoptaecin was upregulated when bees were contaminated with the highest dose of bix. With isolated use of the other two a.i., and the combination of the three a.i. at both doses, hymenoptaecin was downregulated in bees from both feeding management groups, except for bees from the SF group exposed to the lower dose of tri (Figure 2).

The expression of apidaecin was upregulated in bees from both dietary managements, except for those from the SF group exposed to the lower dose. This gene was downregulated with pro and BPT, except for the higher dose of pro. With 7 µg of tri per bee, there was upregulation of apidaecin in bees from the SF group (Figure 2).

Observing the effects of each fungicide on all genes, it was noted that bix promoted a decrease in gene expression only with the lower dose and only for abaecin in the SF group. For bees from the same dietary management exposed to the higher dose of bix, there was a change in the expression of all evaluated genes, with upregulation of all except abaecin, which was downregulated. For bees from the RF group exposed to the lower dose of bix, there was an increase in the expression of six genes (CAT, GPX-1, GST-1, CYP306A1, vitellogenin, defensin-1, and apidaecin). With 7 µg of bix per bee from the RF group, there was upregulation of all genes, except for SOD-1, which was similar to the control, and abaecin, which was downregulated (Figure 2).

Exposure of bees to the lower dose of pro promoted a decrease in the expression of the hymenoptaecin and apidaecin genes in bees from the SF group and of all antimicrobial peptide genes in the RF group. With the higher dose, in the SF group, there was a change in the expression of GST-1, CYP306A1, vitellogenin, defensin-1, and hymenoptaecin. In bees from the RF group exposed to 7 µg of pro, the expression of all genes was altered except defensin-1 and hymenoptaecin (Figure 2).

Tri promoted alteration of expression of six genes in bees from the SF group, with three genes affected by the lower dose (CYP306A1, vitellogenin, and defensin-1) and three by the higher dose (abaecin, hymenoptaecin, and apidaecin). For bees from the RF group, there was a change in the expression of all genes except CAT, GST-1, vitellogenin, and apidaecin, when these bees were individually exposed to 1 µg of tri. With the higher dose of tri, the expression of GPX-1, vitellogenin, defensin-1, and apidaecin was altered in bees from the RF group (Figure 2).

In the bees exposed to the mixture of the three fungicides (BPT), a greater number of gene expression alterations was observed. For bees from the SF group, there was no difference compared with the control (without fungicide) for defensin-1 (with 1 µg) and GST-1 (with 7 µg). For bees from the RF group, there was no alteration in the expression of the genes SOD-1 (with 1 µg), GPX-1 (with 7 µg), and GST-1 (with 7 µg). Considering all comparisons of the treatments within each group with the respective control, it was observed that bees from the RF group showed 50% more gene expression alterations than bees from the SF group.

## 4. Discussion

### 4.1. Colony Feeding Management

The use of pollen traps reduced the availability of this protein food for the bees. The mean total amount of bee-collected pollen per colony during the 15 weeks was 507 g, which was equivalent to 57.9% of the total bee-collected pollen brought into the hives by the bees of this group. This quantity is relatively small considering that up to 20 kg per hive per year can be obtained [41]. Maintaining pollen traps in hives for several weeks can result in a reduction in the quantities of collected pollen due to the population adjustment made by the bees [42]. However, in this study, the amount of bee-collected pollen harvested was relatively small from the early days of food restriction, indicating low availability of pollen in the field [26].

Unlike other livestock, beekeeping does not require routine feeding of colonies, except during periods of heavy rains, severe winters, and scarcity of nectar and pollen in the field [43]. Our study was conducted in an experimental apiary used for several years, with a known natural food flow, which is characterized by low availability of nectar and pollen in most months.

Among the five colonies subjected to food restriction management, one succumbed. The others survived, though with visibly reduced populations, relying on the nectar and pollen foragers managed to bring into the colonies, passing through the pollen traps. For the other group (SF), providing protein paste made of honey and bee-collected pollen, which are foods that bees naturally consume, may have contributed to the acceptance of this paste, which was completely consumed by the bees. Adequate diets can contribute to immunocompetence and resistance to pathogens [44,45] and pesticide tolerance [46]. Bees from the SF group consumed all the energy syrup offered twice a week. This nectar substitute, in association with the protein supplement, may promote a search for more protein food in the field, increased queen egg laying, and worker hygienic behavior [43].

### 4.2. Gene Expression Changes in Bees from Colonies with Supplemented or Reduced Feeding

According to the safety data sheet of the commercial fungicide containing the active ingredients bix, pro, and tri [47], the contact LD_50_ for honey bees is greater than 200 µg per individual. In our study, for treatments with the mixture of all three a.i., the doses used (1 or 7 µg per individual of each a.i.) can be considered sublethal, as they are approximately 10 and four times lower than the lethal dose reported by the manufacturer, respectively.

Christen et al. (2019) [48] evaluated the toxic effect of azoxystrobin, a fungicide belonging to the strobilurin group, which includes tri. They observed downregulation of genes encoding enzymes involved in metabolism, oxidative phosphorylation, and hormonal regulation, which could affect energy production, ontogeny, and behavior of bees. Tri had already been detected in bee-collected pollen obtained from cultivated plants and in bee-collected pollen from nearby wild plants [49]. Bix was detected in samples of bee bread [50], and pro has been detected in bee-collected pollen [51,52,53]. Information regarding bee exposure through contact is limited.

Studying gene expression in bees can aid in understanding the effects of exposure to stressors such as pesticides. In our study, genes related to oxidative stress and detoxification (SOD-1, CAT, GPX-1, GST-1, and CYP306A1), nutrition and longevity (vitellogenin), and immunity (defensin-1, abaecin, hymenoptaecin, and apidaecin) were chosen. The metabolism of bees, like other aerobic species, involves the formation of free radicals. When these molecules are not properly processed to favor cellular homeostasis, oxidative stress can occur [54].

Regulation and inactivation of free radicals are carried out by the antioxidant system. This process is natural due to the metabolism of oxygen and other substances metabolized by bees; however, biotic and abiotic stressors can disrupt cellular homeostasis, generating more reactive oxygen species than the individual can neutralize and eliminate [55,56]. The fungicides chlorothalonil, azoxystrobin, and folpet, when administered to adult bees at sublethal doses in syrup, caused transcriptional alterations in genes encoding enzymes involved in oxidative phosphorylation and metabolism. Among the fungicides, chlorothalonil had the most significant impact, reducing the transcription of genes related to energy production, metabolism, and the endocrine system. Disrupted energy production may decrease foraging activity and cause hormonal imbalances, affecting the transition of nurse bees to foragers [48].

Sublethal doses of insecticides such as organochlorines and organophosphates [57,58] and neonicotinoids [59] may lead to increased production of antioxidant enzymes such as SOD-1, CAT, and GPX, aiming to maintain cellular homeostasis. However, higher pesticide doses and specific characteristics of the xenobiotic can impair the production of these enzymes [60]. Bix and tri are inhibitors of the respiratory chain, and their action may be unfavorable for the production of antioxidant enzymes [61,62].

The gene CYP306A1 belongs to the cytochrome P450 family, which is involved in various cellular biosynthesis and detoxification processes that are particularly important when there is exposure to xenobiotics. This orthologous gene is known for its involvement in ecdysteroid biosynthesis and had its detoxification function described in the silverleaf whitefly *Bemisia tabaci* (Aleyrodidae) exposed to the insecticide imidacloprid [63]. Upregulation of CYP6 genes is associated with resistance to pyrethroids and neonicotinoids in other insects. It is suggested that these genes may be useful for bees to increase detoxification activity when there is pesticide contamination [64]. In our study, expression of the CYP306A1 gene was upregulated with all active ingredients used individually, except for pro and tri at the lowest dose, for bees from the SF and RF groups, respectively.

In another study [65], 121 pesticide contaminants found in bees were tested to determine their effect on the active site of CYP9Q1, a broadly substrate-specific P450 with high quercetin-metabolizing activity that also metabolizes pesticides. Six triazole fungicides were identified, all fungal P450 inhibitors, that fit into the catalytic site. In adults fed combinations of quercetin and the triazole myclobutanil, the expression of five out of six mitochondrion-related nuclear genes was downregulated. Adult bees consuming quercetin with myclobutanil metabolized less quercetin and produced less thoracic ATP. The authors highlight that, although fungicides lack acute toxicity, they may affect bee health by interfering with detoxification by quercetin, compromising mitochondrial function and ATP production.

Vitellogenin is a storage protein, and vitellogenin gene expression is affected by the nutritional state of the individual. It can be considered a pleiotropic gene because, in addition to assisting in lipid transport, it is related to longevity, immunomodulation, and regulation of oxidative stress [66]. In our study, exposure to any one of the three a.i. promoted upregulation of vitellogenin, except for pro at the lowest dose and tri at the highest dose, for bees from the RF group.

Regarding the genes strictly linked to the bees’ immune systems, there was evident alteration in gene expression. There was a change in relative immune gene expression in 63% of the comparisons with the respective control for the SF group of bees and 72% for the RF group. For the other genes, there was a change in expression in 44% and 65% of the comparisons, respectively, for SF and RF. Thus, it is understood that the immune system was more activated in well-fed bees to mitigate the metabolic stress caused by the fungicides, especially when used in a mixture, even at the lowest concentration of each (1 µg). Comparing the gene expression results obtained from bees from colonies subjected to different nutritional managements, it was observed that the mitigating effect from nutritional supplementation was less for genes related to the immune system. However, to substantiate the effects of nutrition on the health of bees exposed to fungicides through contact, additional assessments should be conducted, such as longevity, behavior, and other physiological variables.

Gene expression is a process that occurs at the expense of energy produced in cells. With a need for increased expression of a certain gene for the cell to continue functioning in homeostasis, more energy is demanded [67]. However, even with a higher demand for gene expression so that the harmful effects of xenobiotics do not impair cellular functioning, energy availability can be a determining factor in the response to an intoxication challenge. Of the three a.i. evaluated in this study, bix and tri are known to act as inhibitors of cellular energy production [61,62,68]. With the exposure of bees to these a.i. at the highest evaluated dose, cellular energy demand may not have been met, and thus, gene expression was impaired. As a consequence, bees may become more vulnerable to other stressors, given the fragility resulting from exposure to the fungicide with all three a.i.

The exposure of bees to multiple pesticides simultaneously can result in synergism compared with the effects of the individual active ingredients. The mixture of an insecticide (thiacloprid) with a fungicide (cyproconazole) led to synergistic toxicity in bees, including the expression of the genes CYP306A1, CYP6AS14, apidaecin, defensin-2, and vitellogenin [69]. The response to the challenge with bix, pro, and tri, whether isolated or in combination, was somewhat different among bees that had supplemented versus restricted feeding for most comparisons.

## 5. Conclusions

Bix, pro, tri and the mixture of all three fungicides induced gene expression changes in honey bees when exposed by contact.

Bees from colonies with restricted food availability had gene expression alterations, due to the fungicide treatments, in more genes than bees from colonies with supplemented feeding, especially evident in antioxidant and de-toxification genes. The dose of 7 µg resulted in a greater number of gene expression changes compared with the 1 µg dose, except for the mixture of the three fungicides, which led to a similar number of changes when comparing the two doses for bees from both nutritional management systems.

## Figures and Tables

**Table 1:** A reference gene and genes related to bee health that were analyzed, and their respective primer sequences (GenBank accession numbers) and references.

Genes	Sequence (5′-3′)	References
Ribosomal Protein L32 (RPL32) (former rp49)	F: CGTCATATGTTGCCAACTGGT R: TTGAGCACGTTCAACAATGG	[33]
Superoxide Dismutase 1 (SOD-1)	F: GGTGGTGGTCATTTGAATCATTC R: AAGAAGTGCAGCGTCTGGTTTAC	[34]
Catalase (CAT)	F: TGGAGCAAGTCCTGATAAAATGC R: TGGGCCAAGACGATGTCTATG	[34]
Glutathione Peroxidase 1 (GPX-1)	F: CGACAACTATAAGGAAGCGAAA R: AGATAGAAAAACGTCTTCGCCT	[35]
Glutathione S-transferase 1 (GST-1)	F: TGCCGATCGATTTTTATCAACTT R: AGCCGTCAACGCAACTGC	[35]
Cytochrome P450 306A1 (CYP306A1)	F: CGTCGATGGGAAGGATAAAA R: TCGGTGAAATATCCCGATTC	[35]
Vitellogenin	F: TCGACAACTGCGATCAAAGGA R: TGGTCACCGACGATTGGATG	[36]
Defensin-1	F: TGCGCTGCTAACTGTCTCAG R: AATGGCACTTAACCGAAACG	[37]
Abaecin	F: AGATCTGCACACTCGAGGTCTG R: TCGGATTGAATGGTCCCTGA	[37]
Hymenoptaecin	F: CTCTTCTGTGCCGTTGCATA R: ′GCGTCTCCTGTCATTCCATT	[37]
Apidaecin	F: CTTTGTAGTCGCGGTATTTGG R: AGGCGCGTAGGTCGAGTAG	[37]

## Data Availability

The raw data supporting the conclusions of this article will be made available by the authors on request.
